# Supplementary material for: Considerations for Developing a Reassessment Process: Report from the Canadian Real-World Evidence for Value of Cancer Drugs (CanREValue) Collaboration’s Reassessment and Uptake Working Group
Source: Curr Oncol. 2021 Oct 16;28(5):4174–83. doi: 10.3390/curroncol28050354 (PMC8534602; doi:10.3390/curroncol28050354)
Supplement: Supplementary file 1 [file curroncol-28-00354-s001.zip › curroncol-1378531-supplementary.pdf]

Supplementary Materials

Round 1 Survey Questions

RWE Reassessment and Uptake Working Group Survey 1

1. Who should lead and/or conduct the reassessments?
2. How should reassessments be conducted? (i.e. the same as initial drug reviews, or differently?) If differently than initial drug reviews, how would they be different?
3. What should be reassessed (i.e. clinical data only, clinical and economic, etc.)?
4. What type of data/evidence should be reassessed? How might it be different than the evidence reviewed for initial drug reviews?
5. What are the enablers and barriers to conducting reassessments? (e.g. capacity and resources to put together a submission for a reassessment)
6. What are the enablers and barriers to re-visiting funding decisions?
7. What information would be required in order to re-visit a funding decision?
8. How could/should industry be engaged in the development of this reassessment framework?
9. How could/should industry be engaged in reassessments (e.g. making submissions for reassessments, collecting data, etc.)
10. How should recommendations be made on reassessments? Do they require a different framework, different recommendation categories than for initial drug reviews?
11. How could/should patients and/or patient groups be engaged in the development of this reassessment framework?
12. How could/should patient and/or patient groups be engaged in reassessments (e.g. making submissions for reassessments, collecting data, etc.)
13. What components of the reassessment process should be transparent (e.g. topic selection, timelines, evidence reviewed, recommendations, etc.)
14. What are your expectations of the timelines for reassessments?
    1. From the decision point to collect data to the final reassessment recommendation?
    2. From the initiation of the reassessment to the final reassessment recommendation?
15. Please provide any additional information that you think needs to be considered when developing a framework for reassessment/funding decision.

Note: 13 respondents completed the survey

Round 2 Survey Questions

RWE Reassessment and Uptake Working Group – Survey 2

The following section includes questions where majority (or all) of us previously agree on. These are known as consensus questions, and we would like to get your final approval or identify any remaining concerns related to them.

1. Reassessment should be initiated by (please check all that apply):
   a. Provincial/jurisdictional drug plans or cancer agencies
   b. Industry
   c. Other organizations
      - If others, please specify:

2. Reassessment should be conducted by CADTH/pCODR.
   a. Yes, I agree.
   b. No, I disagree.

c. If you responded no, please indicate who should conduct reassessments

3. All types/sources of data should be considered during the reassessment, as long as the data addresses the uncertainty raised in the initial assessment (or addresses the uncertainty that triggered the reassessment)
   a. Yes, I agree.
   b. No, I disagree.
   c. If you responded no, please indicate which sources of data would be appropriate for reassessments

4. The pCODR Expert Review Committee (pERC) should make recommendations on the reassessments that would be shared with provinces/jurisdictions and cancer agencies
   a. Yes, I agree.
   b. No, I disagree.
   c. If you responded no, please indicate who should provide recommendations for reassessments

5. The recommendation framework for reassessments should be similar to the current deliberative framework for the initial assessment with adaptations to the adoption and feasibility quadrant.
   a. Yes, I agree.
   b. No, I disagree.
   c. If you responded yes, please describe the adaptations you would make to the adoption of feasibility quadrant. If you responded no, please describe what considerations should be included in a deliberative framework

6. The final recommendation categories will be 1) status quo; 2) revisit negotiation; 3) do not recommend.
   a. Yes, I agree.
   b. No, I disagree.
   c. If you responded no, what recommendations category would you add/delete?

7. A 3-6 month timeline is a reasonable timeframe for a reassessment (from the point of initiative a review new data to issuing a recommendation).
   a. Yes, I agree.
   b. No, I disagree.
   c. If you responded no, what is reasonable timeframe for the reassessment

8. Recognizing that it is likely not possible to align the duration of individual provincial Product Listing Agreements (PLAs) across the provinces, the PLAs routinely have a clause that they can renegotiate at any point during the PLA if there is a need for a join renegotiation due to a reassessment.
   a. Yes, I agree.
   b. No, I disagree.
   c. Not applicable
   d. If you responded no, please explain

9. Reassessment should not be applied routinely for all drugs
   a. Yes, I agree – reassessment should be limited to certain drugs
   b. No, I disagree – all drugs should be reassessed
   c. If you responded no, do you have a suggestion how drugs could be prioritized for reassessment?

The following section includes questions where we have received many helpful comments which require additional clarification. These are known as clarifying questions, and we would like to get your additional feedback on.

10. It is anticipated that the majority of reassessment recommendations will be to revisit negotiations, what challenges does this pose to the negotiation process (i.e. pCPA) and the provincial drug plans and cancer agencies

11. Should there be a 'cap' on the number of reassessments conducted per year to manage the volume/capacity? If there should be a "cap", what strategies may be used ot prioritize reassessments?

12. Is it feasible to have 2 pathways for reassessment: short/tailored and comprehensive?
    a. Yes, it is feasible
    b. No, it is not feasible
    c. If you responded yes, please provide an example of a short/tailored reassessment vs a comprehensive reassessment. If you responded no, what might be more feasible

13. Would provinces (drug plans) pay more for a drug if there is rigorous evidence that the drug performs better than the evidence used to inform the initial assessment?
    a. Yes, the drug plan could pay more
    b. No, the drug plan could not pay more
    c. Not applicable
    d. If you responded no, how could this possible scenario be managed with industry?

14. Would provinces (drug plans) be able to delist a drug that is not demonstrating value compared to relevant available treatment option?
    a. Yes, the drug could be delisted
    b. No, the drug could not be delisted
    c. If you responded no, in addition to high quality evidence, is there something else that could support the ability of the drug plans to delist a drug?

15. How can industry be incentivized to participate in reassessments?

Note: 12 respondents completed this survey

Round 3 Survey Questions

RWE Reassessment and Uptake Working Group – Survey 3

1. Reassessments would be conducted for drugs that had received a positive or conditional recommendation from pERC, there would be no reassessments for negative recommendations. If a company wanted to make a resubmission on a negative recommendation, they can do so, as per the existing resubmission procedure.

2. If CADTH were to conduct the reassessments, as per their current procedures, they would only have access to the list (public) prices for the drug undergoing reassessment. From your perspective, is this a substantial concern, and if so, do you have a suggestion as to how an economic evaluation for a reassessment could be conducted using the negotiated (confidential) price?

3. There is agreement among the Working Group that 2 review streams are likely required: 1) a tailored (shorter) review; and 2) a comprehensive (longer) review. As we think about how to decide whether a drug should under a tailored vs a comprehensive review, what do you think of the following proposal: Tailored reviews are for questions related specifically to a focused feasibility issue (dosing, administration schedule, etc.), but would not change the original recommendation. Comprehensive review for questions on the effectiveness (including outcomes of survival, safety, quality of life) and/or cost-effectiveness of a drug, and pERC would make a reassessment recommendation (status quo, revisit negotiation, do not recommend).

4. What other considerations should be included in the decision of tailored vs comprehensive?

5. In relation to question #3, do you think that a cost-effectiveness analysis will always be required for both tailored reviews and comprehensive reviews?
    - Yes for tailored reviews
    - No for tailored reviews
    - Yes for comprehensive reviews
    - No for comprehensive reviews
    - Please explain your selection

6. What information is required to revisit a funding decision in addition to the pivotal trial? For instance, would data on utilization, updated cost-effectiveness analyses, or real-world data on important outcomes (survival, safety, quality of life) be required? Please provide details on what information you think would be required for a funding decision to be revisited?

7. Could CADTH's existing jurisdictional working groups (e.g., Provincial Advisory Group, PAG) be used for the purposes of selecting and prioritizing drugs for reassessment? What other mechanisms could be put in place to prioritize reassessments?

8. Should there be a cap on the number of reassessments conducted per year?

Note: 10 respondents completed this survey

Round 4 Survey Questions

RWE Reassessment and Uptake Working Group – Survey 4

Case scenario: For bevacizumab, the WG members were divided between status quo and revisit negotiations. In this example, the recommendation is complicated by the entrance of a biosimilar. Members who suggested status quo felt that renegotiating Avastin again is unlikely and given that there no alternative for Avastin, it might be practical to wait for the entrance of the biosimilar. However, some members felt that issuing a status quo recommendation for Avastin might impede pricing negotiation for the biosimilar product. Given that Avastin has a high budget impact and the ICER is relatively high, it would make sense to revisit the pricing and renegotiate.

Based on this case example, members felt a need for more subtle definition for the reassessment categories. How would you modify the recommendation categories?

1. What are some potential challenges you anticipate with the implementation of RWE to revisit a funding decision, price renegotiation, and/or delisting a drug?
    a. Revisit a funding decision (this could mean expanding or restricting criteria)
    b. Renegotiating the price

c. Delisting a drug

2. What are some potential facilitators and solutions that can support the use of RWE to revisit a funding decision, price renegotiation, and/or delisting a drug?
   a. Revisit a funding decision (this could mean expanding or restricting criteria)
   b. Renegotiating the price
   c. Delisting a drug

3. Have you or your organization reassessed a funding decision and/or changed listing or funding criteria or delisted a drug? What lead to this reassessment? If you answered yes to question 1, what were some challenges you experienced with respect to revisiting a funding decision and/or changing the listing or delisting a drug?
   a. What were some solutions or facilitators that made this process easier and/or more successful?

4. Are there other areas, fields or jurisdictions that we should be learning from (both success and failure)? Why did they succeed or fail?

5. What important factors will be necessary to ensure the successful implementation of any framework developed?
   a. What may lead to its failed implementation??
   b. Which stakeholders need to be aligned?

Note: 14 respondents completed this survey

Stakeholder Consultation: Questions for Consideration

1. What barriers do you foresee in the implementation of such a framework that is proposed? What potential solutions/facilitators would ensure proper implementation.
   a. In terms of the implementation of this framework: who should participate in the reassessment of a drug that is currently publicly funded?
   b. What role should each stakeholder have in the reassessment process?
   c. In an ideal scenario, what do you foresee your role being in the process to reassessment a drug that is currently publicly funded?
   d. How should the results from a reassessment be disseminated to different stakeholders or the public?
   e. Should different criteria be used to re-assess a drug compared to when it is assessed for the first time? If so, which criteria should be different?

2. What benefit/opportunities do you anticipate for your organization or the healthcare system if there was a mechanism to re-review a drug that is currently publicly funded?

3. As we are in the planning stage of the CanREValue proposal for a reassessment process, we welcome collaboration and engagement from interested stakeholders. Please indicate how you would like to be involved in the development and implementation of this proposal.

Summary of Respondents for the Stakeholder Consultation

Category	Total Number of Feedback Reports Received (N = 21)	% of All Feedback Reports Received
Academic/Researcher	3	14.3%
Individual Pharmaceutical Company	10	47.6%
Industry-related organization	3	14.3%
Patient Group	4	19.1%
Other	1	4.7%
